# From Clinical Suspicion to Diagnosis: A Review of Diagnostic Approaches and Challenges in Fungal Keratitis

**DOI:** 10.3390/jcm13010286

**Published:** 2024-01-04

**Authors:** Panagiotis Toumasis, Andreas G. Tsantes, Anastasia Tsiogka, George Samonis, Georgia Vrioni

**Affiliations:** 1 Department of Ophthalmology, 212 Military Hospital of Xanthi, 67100 Xanthi, Greece; 2 Department of Microbiology, Saint Savvas Oncology Hospital, 11522 Athens, Greece; 3 Laboratory of Haematology and Blood Bank Unit, Attikon Hospital, School of Medicine, National and Kapodistrian University of Athens, 12462 Athens, Greece; 4 First Department of Ophthalmology, National and Kapodistrian University of Athens, “G. Gennimatas” General Hospital, 11527 Athens, Greece; 5 Department of Medicine, University of Crete, 71500 Heraklion, Greece; 6 Department of Microbiology, Medical School, National and Kapodistrian University of Athens, 11527 Athens, Greece

**Keywords:** fungal keratitis, fungal ocular infections, diagnosis

## Abstract

Fungal keratitis is a relatively rare yet severe ocular infection that can lead to profound vision impairment and even permanent vision loss. Rapid and accurate diagnosis plays a crucial role in the effective management of the disease. A patient’s history establishes the initial clinical suspicion since it can provide valuable clues to potential predisposing factors and sources of fungal exposure. Regarding the evaluation of the observed symptoms, they are not exclusive to fungal keratitis, but their timeline can aid in distinguishing fungal keratitis from other conditions. Thorough clinical examination of the affected eye with a slit-lamp microscope guides diagnosis because some clinical features are valuable predictors of fungal keratitis. Definitive diagnosis is established through appropriate microbiological investigations. Direct microscopic examination of corneal scrapings or biopsy specimens can assist in the presumptive diagnosis of fungal keratitis, but culture remains the gold standard for diagnosing fungal keratitis. Advanced molecular techniques such as PCR and MALDI-ToF MS are explored for their rapid and sensitive diagnostic capabilities. Non-invasive techniques like in vivo confocal microscopy (IVCM) and optical coherence tomography (OCT) are useful for real-time imaging. Every diagnostic technique has both advantages and drawbacks. Also, the selection of a diagnostic approach can depend on various factors, including the specific clinical context, the availability of resources, and the proficiency of healthcare personnel.

## 1. Introduction

Fungal keratitis, also referred to as mycotic keratitis or keratomycosis, stands as a relatively rare yet severe corneal infection. It is triggered by an array of fungal species—the most commonly implicated ones being *Fusarium* spp., followed by *Aspergillus* spp., and *Candida* spp. [1]. It is estimated that a minimum of 1 million cases of fungal keratitis occur every year, with the highest rates in Asia and Africa [1]. Fungal keratitis presents a formidable clinical challenge in the field of ophthalmology, given its potential to yield serious complications, if not promptly diagnosed and treated. The first documented case of fungal keratitis can be traced back to 1879 when German ophthalmologist Theodor Leber described an infection in a 54-year-old farmer who sustained an eye trauma while handling wheat-cutting blades. Since then, the comprehension of fungal keratitis has evolved considerably. Innovative advances in diagnostic techniques and treatment alternatives have paved the way for enhanced patient outcomes. Notwithstanding these advancements, the intricacy of the disease endures, as it can present with diverse clinical manifestations and is attributed to an assortment of fungal species. 

Fungal keratitis presents with a spectrum of potential complications that can irreparably affect eye health [2]. They encompass a range of severity. In addition to the usual symptoms of an eye infection, such as redness, intense eye pain, blurred vision, photophobia, excessive tearing, οr discharge, many patients develop corneal ulcers, i.e., open sores on the cornea, which can lead to corneal melting or perforation. The infection’s progression might elevate intraocular pressure, contributing to secondary glaucoma and potential optic nerve damage. In severe cases, the infection may extend to the inner coats of the eye, causing endophthalmitis, or even worse, the infection may encompass the entirety of the structures of the eyeball, causing panophthalmitis. The worst-case scenario includes profound vision impairment and even permanent vision loss. In low-income and middle-income countries, an annual incidence of approximately 600,000 eyes losing vision due to fungal keratitis is anticipated [1]. Approximately 100,000 eyes are likely removed each year due to delayed diagnosis and poor treatment outcomes [1].

Under threat of the above complications, the precise and prompt diagnosis of fungal keratitis plays a pivotal role in the successful management of this disease, especially in low- and middle-income countries (LMICs) where the prevalence of fungal keratitis is highest and patients may delay presenting to an ophthalmologist. Achieving this objective requires a comprehensive approach that blends clinical assessment, microbiological examinations, and advanced imaging techniques. The expertise to interpret these results effectively is paramount for ophthalmologists. Even though fungal keratitis is a relatively infrequent ocular condition, possessing adept reflexes and the right training is paramount, as ophthalmologists might unexpectedly encounter cases within their daily clinical practice. It is the fusion of diagnostic acumen and proactive preparedness that equips ophthalmologists to navigate the challenges posed by this ailment and provide optimal care to their patients.

The differential diagnosis of fungal keratitis involves distinguishing it from other forms of infectious keratitis that may exhibit similar signs but often respond to different treatment regimens. The differential diagnosis of fungal keratitis includes bacterial keratitis, acanthamoeba keratitis, and necrotizing herpetic keratitis [2]. 

**Figure 1:**
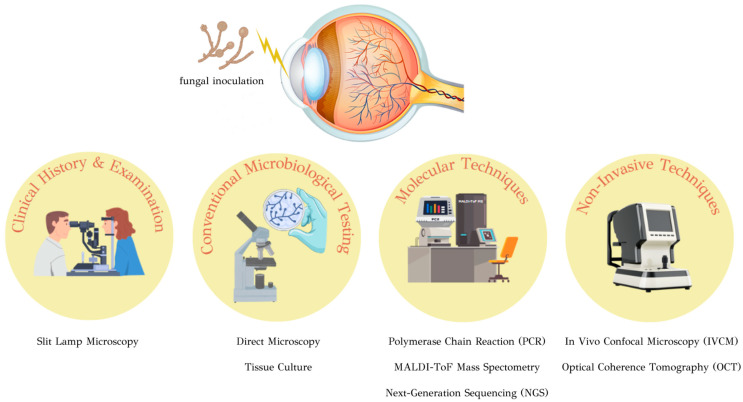
Diagnostic approaches to fungal keratitis.

The purpose of this review is to provide an overview of the journey from initial clinical suspicion to reaching a definitive diagnosis. (Figure 1) Through a meticulous exploration of contemporary literature and recent advancements in diagnostic techniques, this review endeavors to provide a detailed analysis of each approach’s strengths and limitations. These insights are expected to foster a deeper comprehension of the practical applications of diagnostic methods in clinical settings.

## 2. Clinical History

While a definitive diagnosis is often established through appropriate microbiological investigations, a patient’s history and clinical examination findings establish the initial clinical suspicion. The diagnosis of fungal keratitis starts with a strong clinical suspicion. 

A thorough clinical history provides valuable clues regarding potential predisposing factors and sources of fungal exposure. The most common risk factor for fungal keratitis is eye trauma, especially an injury involving plant material or organic debris [3]. A patient with a history of trauma occurring during agricultural practices might lead to direct inoculation with fungal conidia present in the environment. In terms of occupation as a risk factor, individuals engaged in agricultural occupations are at a higher risk of occupational ocular injuries, which, consequently, increases their susceptibility to developing fungal keratitis [3]. Another common risk factor, especially in industrialized countries, is contact lens usage when combined with poor hygiene, extended wear, and improper lens care [3]. Other factors that can be blamed for predisposing to the manifestation of fungal keratitis are: age (patients with acanthamoeba keratitis tend to be younger than those with fungal or bacterial keratitis while bacterial keratitis is more likely to occur in older patients) [4], immunosuppression, like HIV/AIDS or diabetes, pre-existing ocular surface disease, like blepharitis or conjunctivitis, topical corticosteroid use, and previous ocular surgery [3]. 

It is crucial to emphasize that the symptoms observed are not exclusive to fungal keratitis; they can be encountered in different types of infectious keratitis. Common symptoms of fungal keratitis include redness, intense eye pain, blurred vision, light sensitivity (photophobia), the sensation of a foreign body in the eye, excessive tearing, and sometimes discharge [5]. However, extracting details about the onset and evolution of symptoms could provide a comprehensive context that may aid in distinguishing fungal keratitis from other conditions. The duration of symptoms in fungal infection tends to be more prolonged [6], which is a fact that can aid in differentiating fungal keratitis from other conditions with the same clinical presentations, such as bacterial keratitis. 

## 3. Clinical Examination 

Thorough clinical examination of the affected eye can assist in making a diagnosis before microbiological testing or in its absence. Concerning the latter fact, most ophthalmologists in low- and middle-income countries (LMICs) do not have access to specialized ocular microbiological laboratory facilities and may have to rely on their own clinical acumen for diagnosis. Ophthalmologists, by examining the cornea using a slit-lamp microscope, should evaluate the presence of signs of fungal keratitis and think about conditions that could mimic fungal keratitis. In cases of keratitis, there is not a single clinical feature that can be definitively indicative of a specific causative agent. Thus, differentiating fungal keratitis from other types of microbial keratitis can pose a difficulty. In terms of numbers, a study involving 15 ophthalmologists assessing microbiological causes found fungal keratitis to be the most challenging to diagnose. They achieved a sensitivity of 38% and a specificity of 45% [7]. In a separate study using corneal photographs, specialists correctly distinguished between fungal and bacterial keratitis in only 66% of cases [8]. The differential diagnosis of fungal keratitis includes bacterial keratitis, acanthamoeba keratitis, and necrotizing herpetic keratitis [2].

**Figure 2:**
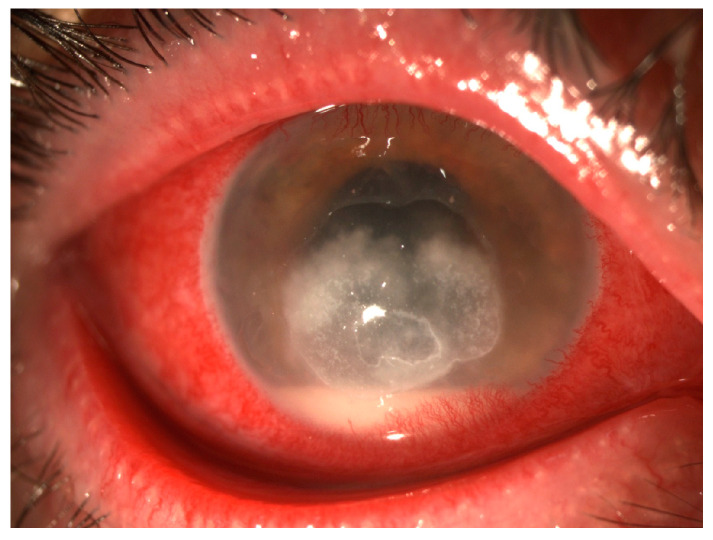
Fungal keratitis—Gross torch light assessment. Big, central, corneal ulcer with serrated margins and epithelial defect, with injection and hypopyon.

**Figure 3:**
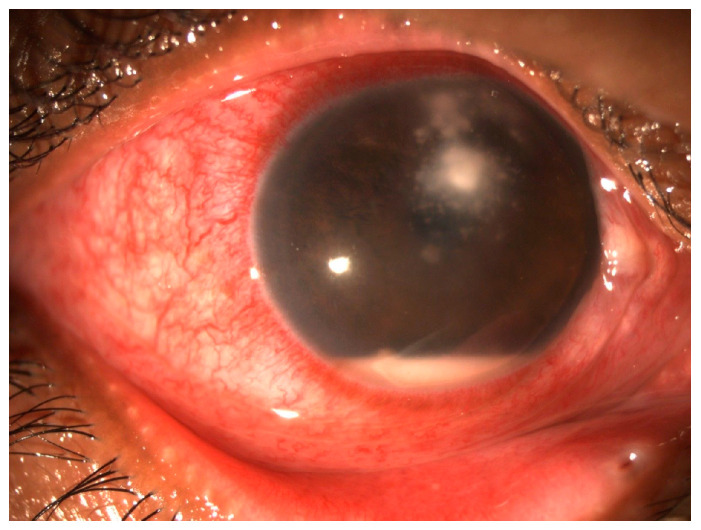
Fungal keratitis—Gross torch light assessment. Corneal ulcer with feathery margins and satellite lesions with injection and hypopyon.

Some clinical features could be valuable predictors for fungal keratitis. (Table 1) (Figure 2 and Figure 3). Elevated edges, branching ulcers, feathery margins, rough texture, and satellite lesions are features suggestive of fungal keratitis [2]. Serrated margins, raised slough, and non-yellow coloration of corneal ulcers have been independently associated with fungal keratitis, whilst the presence of anterior chamber fibrin has been independently associated with bacterial keratitis [9]. By employing an algorithm that relies solely on clinical signs to predict fungal keratitis and omits color as a distinguishing factor, the probability of fungal keratitis stands at a robust 89% when serrated margins, raised slough, and the absence of anterior chamber fibrin are observed [10]. Even though the color of corneal ulcer is a subjective factor, it can sometimes aid in distinguishing the causative agent; dematiaceous molds are known for their dark pigmentation, and when they cause fungal keratitis, they can impart a brown or black color to the corneal ulceration [11,12].

**Figure 4:**
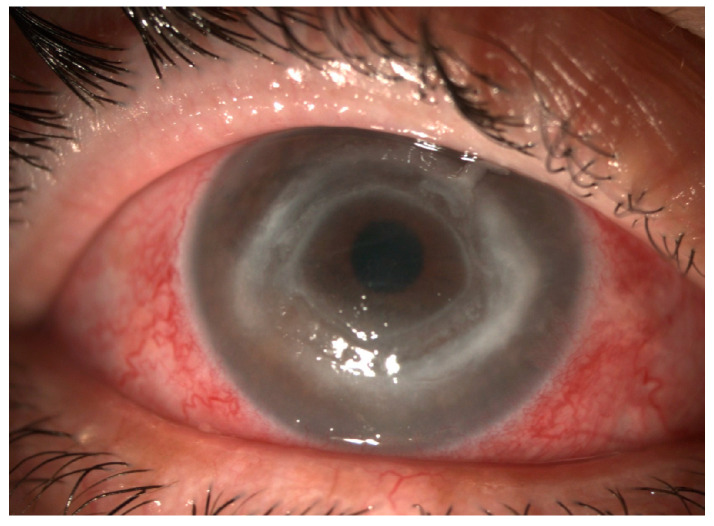
Fungal keratitis—Gross torch light assessment. Wessley immune ring.

There are some clinical features that aid ophthalmologists in distinguishing fungal keratitis from acanthamoeba keratitis. Ring infiltrates occur in fungal and bacterial keratitis (Figure 4), but this is more likely to indicate acanthamoeba keratitis [4]. Secondly, disease confined to the epithelium is more common in acanthamoeba keratitis than fungal keratitis [4]. Also, satellite lesions are observed in both acanthamoeba and fungal keratitis [4], challenging the notion that these lesions are an exclusive characteristic of fungal keratitis [9]. 

Ιn instances of heightened clinical suspicion of fungal keratitis, certain clinical signs may provide indications of a specific fungus as the causative agent. *Fusarium*-induced corneal ulcers often exhibit serrated margins and a corneal infiltrate that is not yellow in color, whereas *Aspergillus*-induced corneal ulcers tend to feature an elevated surface, ring infiltrates, hypopyon, and endothelial plaques [12,13].

## 4. Conventional Microbiological Tests

In cases where mycotic keratitis is strongly suspected, the diagnostic algorithm proceeds with microbiological investigations aimed at identifying the specific causative agent. First of all, clinical sample collection should be performed. 

### 4.1. Sample Collection

Collecting clinical samples is a procedure of paramount importance, involving not only the cornea but also other eye structures. Clinical samples collected for laboratory diagnosis primarily consist of corneal scrapings [2]. The collection of corneal scrapings is performed while the patient is positioned at the slit-lamp, using a Kimura spatula, Bard–Parker knife, sterile razor, surgical blade, or spatula, while local anesthetic eye drops are instilled to the affected eye to minimize ocular discomfort and facilitate the corneal scraping procedure [6]. Ophthalmologists should keep in mind that while scraping helps to remove necrotic tissue, a study has cautioned against overzealous scraping due to the potential for scarring and a subsequent decline in visual acuity [14].

Because fungi have a propensity to infiltrate the deeper layers of the cornea, relying solely on a mere tissue swab is frequently inadequate to verify a fungal infection [15]. Thus, the utilization of deep corneal scrapings becomes necessary [15]. Thus, in certain cases, where deep infection is suspected or corneal scrapings do not yield positive results, more invasive procedures may be necessary [16]. Corneal biopsy is an invasive procedure requiring a minor operation in theatre and should be considered when there is a high clinical suspicion of fungal keratitis, two consecutive negative smear and culture reports, and no observed clinical improvement with empirical antibiotic therapy [17]. In addition to microbiological examinations, the biopsy material can also be sent for histopathological examination [17]. Anterior chamber aspiration should be performed if there are signs indicating the infection has extended beyond the superficial layers of the cornea or in cases with progressive corneal damage and persistent hypopyon [17].

Moreover, it is essential to collect samples from both the same-side (ipsilateral) and opposite-side (contralateral) eyelids and conjunctiva. This is necessary to confirm that the microorganisms identified in the cornea did not come from the temporary and harmless fungal microbiota, often known as the ‘mycobiota’, found in the conjunctival sac [6]. 

It is recommended that all suspected microbial keratitis be scraped for smear and cultures before initiating antibiotic treatment [18].

### 4.2. Microscopy

Direct microscopic examination of corneal scrapings or biopsy specimens can assist in rapid and cost-effective presumptive diagnosis of fungal keratitis. Microscopy enables the direct visualization of fungal structures, such as hyphae or yeast forms, and, by extension, prompt initiation of targeted antifungal therapy, which is crucial in preventing the progression of the infection. Also, in regions with a high incidence of FK, direct microscopy is often the only available diagnostic tool. However, the accurate recognition of fungal structures, and even more so, the identification at the genus/species level, largely relies on the observer’s experience. 

A recommended set of smears for direct microscopic evaluation could be a wet preparation using potassium hydroxide (KOH) stain, ink-KOH, or lactophenol cotton blue (LCB) stain, a smear stained by the Gram or Giemsa method, and a smear stained with special fungal stains such as the Grocott methenamine silver-nitrate (GMS) stain, periodic acid-Schiff (PAS) stain, or Calcofluor white (CFW) [19]. Each stain has its advantages and disadvantages, and the choice often depends on the clinical context and laboratory resources. 

Calcofluor-white (CFW) with fluorescence microscopy seems to be a more sensitive technique than potassium hydroxide (KOH) for diagnosing fungal keratitis, although they share the same specificity. KOH, in turn, is more sensitive than Gram’s stain and lactophenol cotton blue (LPCB) [20]. Calcofluor white (CFW) is regarded as a key component in the diagnostic process. Its sensitivity, when used in conjunction with KOH or Giemsa stain, has been demonstrated to be 96.6% to 98.3%, respectively [21]. Giemsa stain has the ability to detect bacterial or mixed infections [21]. Methylene blue (MB) emerges as a promising stain. A study revealed that it exhibits greater sensitivity and specificity than KOH, yet it still falls short when compared to CFW [22].

### 4.3. Culture

While direct microscopy provides a rapid result to clinicians for starting initial therapy, the culture of corneal samples remains the gold standard for diagnosis of fungal keratitis. Corneal samples, acquired via scraping or biopsy, are inoculated onto culture plates in the shape of multiple ‘C’s. Significance is attributed to growth occurring exclusively within the confines of the C-shaped streaks, whereas growth outside these streaks is considered to be contamination [6,23]. ‘C’-streaks is a standardized method ensuring that any fungal organisms present on the swab have enough space to proliferate and form visible colonies on the culture medium. 

The types of solid culture media that are commonly used for the diagnosis of fungal keratitis are blood agar (BA), preferably sheep blood agar, which should be incubated at 25 and 37 °C, and Sabouraud dextrose agar (SDA), with an incubation temperature at 25 °C [6]. SDA, with its lower pH, aids fungal isolation over bacteria and facilitates identification through spore and pigment enhancement [18]. For corneal fungal pathogens, SDA with chloramphenicol or gentamicin, excluding cycloheximide, is preferred [18]. The omission of cycloheximide in standard SDA in ocular labs prevents the inhibition of saprophytic fungi, which are rarely the cause of fungal keratitis. Additional culture media that have demonstrated effectiveness in the initial isolation of ocular fungi comprise chocolate agar (CA), cystine tryptone agar, and Rose Bengal agar. In a study in India, where resources and cost-effectiveness are significant considerations, comparison between BA, CA, and SDA in terms of the time required for culture growth and cost demonstrated that BA and CA support the growth of all fungi commonly associated with fungal keratitis, and the time taken for growth is shorter than SDA [18]. Although SDA is the preferred standard, the percentage of success in growing fungal elements was slightly better on BA (56%) and CA (46%) compared to SDA (43%) in this study [18]. Also, SDA may be unnecessary for diagnosing fungal keratitis, as certain fungal species that can grow on this medium, like Histoplasma, are not typically causative agents of this condition [18]. 

Liquid culture media can be contemplated, including brain–heart infusion (BHI) broth, incubated at 25°C, which may be the most suitable single medium, especially in cases where there is limited corneal material available [6]. Also, the use of liquid phase media reduces the effect of previous treatment with antimicrobial drugs [24]. If corneal infection with Acanthamoeba sp. is suspected, non-nutrient agar (NNA) should be used [24].

While culture remains the gold standard for fungal keratitis diagnosis, it is important to note that it may take several days to yield results. Initial growth occurs within 72 h in 83% of cultures and within 1 week in 97% of cultures [15]. However, it is worth noting that culture media may necessitate incubation for an extended period, sometimes reaching up to 4–6 weeks [6]. This delay can be a significant drawback in cases where prompt treatment is crucial to preserve vision. Fungal cultures may bring in false-negative results, particularly if the specimen is inadequately collected or if the fungal load is low. This drawback can lead to delayed or missed diagnoses.

## 5. Advanced Molecular Techniques

The significant limitations associated with conventional microbiological investigations have prompted the advancement of molecular techniques as diagnostic tools for fungal keratitis. 

### 5.1. PCR

PCR (polymerase chain reaction) is a powerful diagnostic tool that works by amplifying a specific segment of DNA, making it easier to detect and analyze [25]. Its core principles involve cycles of denaturation, annealing, and extension [25]. During denaturation, DNA melts into two single strands. In annealing, short DNA primers bind to target sequences. Then, a heat-stable DNA polymerase enzyme extends the primers, creating two new DNA strands. This process repeats, exponentially increasing the target DNA. Studies on diagnosing fungal keratitis by the PCR technique have predominantly focused on amplifying a specific segment of the rRNA gene. This preference arises because rRNA genes exhibit significant uniformity across various fungal species. The utilization of rRNA genes for identifying fungal species relies on detecting shared sequences within these rDNA genes. Typically, the target DNA regions include 18S rRNA, 28S rRNA, or the ITSs-5.8S rRNA region, situated between the 18S rRNA and 28S rRNA regions.

PCR can be conducted on diverse clinical samples, such as corneal scrapings/biopsies, tears, or aqueous humors, requiring only a small sample volume [2]. Τhis method allows for the detection of even trace amounts of fungal DNA, increasing the accuracy of diagnosis, especially in cases with a low fungal burden. Thus, PCR could help to establish an early diagnosis.

Moreover, multiplex PCR assays are available, capable of detecting multiple fungal species simultaneously, aiding in differential diagnosis.

Studies have demonstrated that PCR can provide results relatively quickly, often within a few hours, and exhibits a high sensitivity in comparison to conventional microbiological investigations, namely direct microscopy and culture methods [26,27]. However, it might exhibit a reduced specificity, potentially yielding false-positive outcomes, which could be attributed to the amplification of non-pathogenic organisms present in the sample [2].

The speed and accuracy of PCR advocate for its widespread application in the diagnosis of fungal keratitis [26]. It should become a routine part of laboratory testing, alongside staining and culturing, to diagnose fungal keratitis whenever clinicians suspect its presence. However, PCR equipment and reagents can be too expensive, making it less affordable for routine use in LMICs, where the incidence of fungal keratitis is high [28]. 

### 5.2. MALDI-ToF MS

Matrix-assisted laser desorption/ionization time of flight mass spectrometry (MALDI-ToF MS) has emerged as a breakthrough technique and has been successfully applied in recent years in the field of clinical microbiology for the identification of microorganisms. MALDI-ToF MS Z proves to be a valuable method for quickly diagnosing fungal keratitis, especially for cases involving rare or uncommon fungi [29,30,31,32,33,34,35,36].

MALDI-TOF MS is an analytical technique that leverages the principles of laser-induced ionization, precise mass measurement, and spectral analysis [37]. The sample intended for analysis, which can be corneal scrapings, corneal biopsies, corneal smears, corneal button tissue, or contact lens material, is prepared by mixing or coating with a solution of an energy-absorbent, organic compound known as the matrix. This mixture is then applied to a target plate and allowed to dry. Next, a pulsed laser beam impinges on the dried mixture, triggering the ablation and desorption of the sample and matrix material. The matrix absorbs the laser energy, causing it to vaporize and release the sample molecules as ions into the gas phase. These ions are then accelerated through an electric field, with lighter ions traveling faster than heavier ones. The time taken for each ion to reach a detector at the end of a flight tube, called the TOF (time of flight), is directly proportional to its mass-to-charge ratio (*m*/*z*). The resulting mass spectrum, called the PMF (peptide mass fingerprint) displays these *m*/*z* values on the x-axis and ion intensity on the y-axis. Microbial identification using MALDI-TOF MS involves either comparing the PMF of an unidentified organism to the PMFs stored in the database or aligning the mass values of biomarkers from the unknown organism with the proteome database.

MALDI-ToF MS has several advantages as a diagnostic method for fungal keratitis [38]. It offers a swift turnaround time and serves as a reliable diagnostic method, exhibiting both high sensitivity and specificity [38]. Nevertheless, it must be emphasized that there is a lack of published research that directly compares the effectiveness of MALDI-ToF MS with conventional techniques in the diagnosis of fungal keratitis. Only one study that has conducted a comparison between MALDI-ToF MS and traditional approaches like morphology and PCR sequencing encompassed a sample of *Aspergillus* keratitis and revealed a noteworthy level of concurrence among the various diagnostic methods [39]. MALDI-ToF MS is also easy to apply and therefore does not require highly specialized personnel. Furthermore, it is an economic approach, disregarding the initial expense incurred during the purchase of the system, as the cost of consumables remains minimal [38]. The aforementioned points indicate that MALDI-TOF MS is well-suited for tertiary referral centers in low- and middle-income countries (LMICs) where the prevalence of fungal keratitis is highest. 

MALDI-ToF MS might have difficulty distinguishing closely related species, leading to challenges in accurately identifying organisms with similar mass spectra [38]. Additionally, incomplete or outdated databases can result in misidentifications or failures to identify certain species, since accurate identification using MALDI-ToF MS relies on reference databases. Despite these disadvantages, MALDI-ToF MS continues to be a valuable asset for diagnosing fungal keratitis. 

Over the last several years, a new molecular modality, next-generation sequencing (NGS), has gained ground in the diagnosis of fungal keratitis since it provides rapid and precise identification of causative fungi, guiding targeted antifungal therapy [40,41]. Ιt seems that NGS has a higher sensitivity than that reported for aerobic culture [41]. However, additional testing is required to ascertain the clinical significance of extra organisms identified by NGS in infected cases, along with those isolated from normal corneas [41].

## 6. Non-Invasive Diagnostic Techniques 

While traditional diagnostic methods continue to be significant, non-invasive diagnostic techniques are increasingly gaining prominence in the diagnostic algorithm. This is primarily because they can swiftly identify the causative agent in real time. Non-invasive methods of diagnosis include in vivo confocal microscopy (IVCM) and optical coherence tomography (OCT). 

### 6.1. In Vivo Confocal Microscopy (IVCM)

This is a non-invasive imaging technique that enables real-time morphological analysis of all corneal layers and their micro-anatomic structures (cells, nuclei, and nerves). It provides magnifications ranging from 200 to 500, offering enhanced image contrast and the ability to visualize details even in hazy corneas [42]. This technology supports repeated observations of fungal elements, such as hyphae and spores, assisting ophthalmologists in the diagnosis, management, and follow-up of cases of fungal keratitis [42]. The sensitivity of IVCM varies up to 94%, while the specificity is up to 92% [42,43,44,45].

IVCM has certain limitations. IVCM cannot be used to distinguish between the principal types of causative fungi (*Aspergillus* spp. and *Fusarium* spp.) on the basis of their different branching angles, and culture remains essential to determine fungal species [44,46]. It is worth mentioning that while IVCM has shown its effectiveness in diagnosing fungal and acanthamoeba keratitis, its resolution constraints prevent the confirmation of bacterial infections, as bacteria are too small to be observed [42,47]. Although IVCM is considered a non-invasive technique, it is, in fact, a contact diagnostic tool [47]. It requires patient cooperation and the ability to keep the eye still during the examination to obtain high-quality images and to carry out a dynamic examination [47]. This requirement may pose challenges for some patients, especially children or individuals experiencing eye discomfort [47]. Moreover, the quality of IVCM images relies heavily on the expertise of the operator. Inexperienced operators may have difficulty obtaining clear images, potentially leading to misinterpretations [45,47]. Also, IVCM equipment can be expensive, and its availability may be limited to a few specialized ophthalmology centers in high-income countries. 

### 6.2. Optical Coherence Tomography (OCT)

Optical coherence tomography (OCT) utilizes low-coherence interferometry to create cross-sectional images of the cornea, allowing ophthalmologists to assess its thickness and detect the presence of infiltrates [48]. In cases of fungal keratitis, OCT can detect changes in the cornea typical for the mycotic process [49]. In the initial phases of microbial keratitis, the cornea exhibits thickening in the infiltrated region. Both the epithelium and the endothelium typically appear as hyperreflective layers when compared to the stroma. Edema manifests as a diffuse thickening of the stroma, resulting in changes in the curvature of the posterior corneal surface. As the infection and inflammation resolve, the degree of corneal thickening diminishes. In advanced stages, patients often develop scarring, causing the affected cornea to become thinner than the surrounding healthy areas due to the retraction of scar tissue. Notably, OCT features distinctive to aggressive fungal keratitis encompass the presence of limited cystic formations of varying sizes within the stroma, representing necrotic tissue.

By utilizing OCT and confocal microscopy, it becomes possible to visualize a huge percentage of endothelial plaques that are characteristic of fungal keratitis [50]. OCT is more commonly employed compared to confocal microscopy. While the observable corneal damage provides only indirect indications of the presence of the fungal pathogen, precluding species identification, the OCT method offers convenience in assessing the corneal condition over time and facilitates the tracking of changes across the entire cornea.

## 7. Conclusions 

In conclusion, the diagnosis of fungal keratitis represents a crucial aspect in the effective management of this potentially vision-threatening condition. Over the years, conventional microbiological methods, namely direct microscopy and culture, have served as valuable tools but the landscape of diagnosis is continually evolving with the introduction of advanced molecular techniques, substantially bolstering our capacity to rapidly and accurately identify the responsible fungal species. 

**Figure 5:**
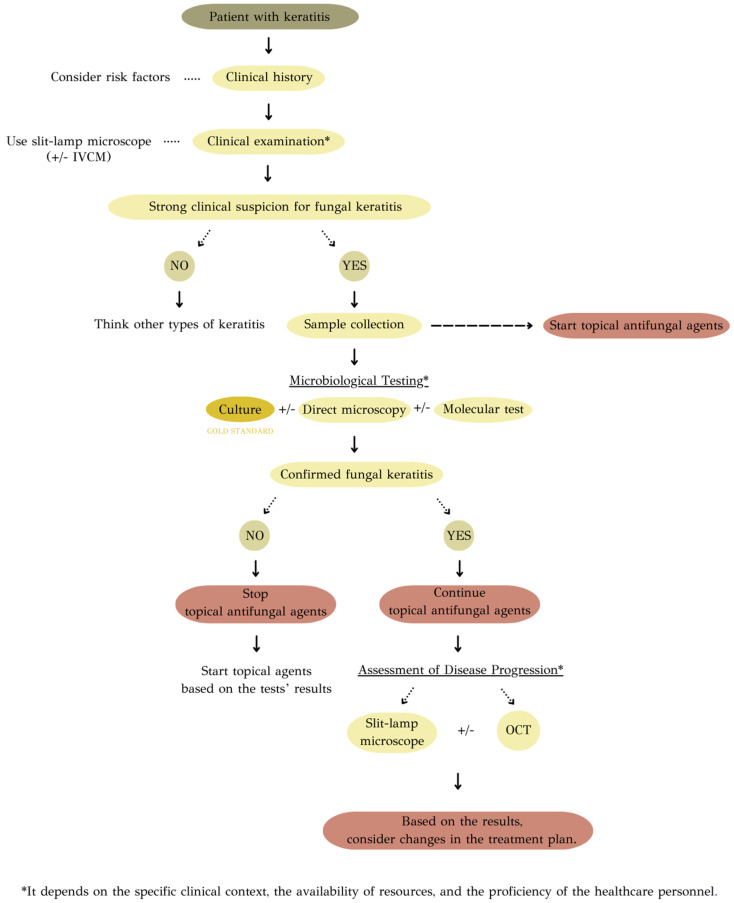
Recommended diagnostic algorithm of fungal keratitis.

Herein, a diagnostic algorithm is recommended. It should be emphasized that in cases of strong clinical suspicion of fungal keratitis, antifungal therapy should be initiated promptly, which can be modified based on the results of various tests conducted (Figure 5).

It is important to acknowledge that every diagnostic technique has both advantages and drawbacks (Table 2). Also, the selection of a diagnostic approach can be contingent upon various factors, including the specific clinical context, the availability of resources, and the proficiency of the healthcare personnel. In many cases, a multidisciplinary approach, drawing upon the expertise of ophthalmologists, microbiologists, and other specialists, becomes indispensable. Such collaboration ensures a comprehensive evaluation and the most effective strategies for patient management.

## Figures and Tables

**Table 1:** Slit-lamp microscope findings indicative of fungal keratitis.

Elevated edges Branching ulcers Feathery/serrated margins Rough texture Satellite lesions Non-yellow coloration

**Table 2:** Advantages and drawbacks of different diagnostic approaches to fungal keratitis.

Sample	Sample Harvesting	Method	Findings	Comments
Corneal sample	Corneal scrapping or corneal biopsy	Tissue culture	Growth on C-streaks in culture media (BA, SDA, CA)	Gold standard Sampling from ipsilateral and contralateral eyelids and conjunctiva to rule out benign mycobiota Positive within 1 week in 97% of cases Sensitivity: About 60% [51]
Corneal sample	Corneal scrapping or corneal biopsy	Histopathology (Direct Microscopy)	Visualization of fungal structures, such as hyphae or yeast forms (KOH stain, CFW stain)	96.6–98.3% sensitivity of CFW stain combined with KOH or Giemsa stain
Corneal sample	Corneal scrapings, corneal biopsies, corneal smears	PCR	rRNA genes (18S, 28S ITSs-5.8S)	Quick results often within a few hours Sensitivity: Up to 94% [51]
Corneal sample, contact lens material	Corneal scrapings, corneal biopsies, corneal smears, corneal button tissue	MALDI-ToF MS	PMF of fungal microorganism	Quick turnaround time Sensitivity: Up to 97% [51] Economic approach Does not require highly specialized personnel. Difficulty distinguishing closely related species with similar mass spectra
Non-invasive	Non-invasive	In vivo confocal microscopy	Direct visualization of fungal elements such as hyphae and spores	Sensitivity up to 94%, specificity is up to 92% [42,43,44,45] Does not distinguish between the principal types of causative fungi Discomfort for patient Expensive equipment—Not readily available
Non-invasive	Non-invasive	Optical coherence tomography	Initial stage: thickened cornea, hyperreflective epithelium/endothelium, cystic formations within stroma Advanced stage: scarring resulting in thinner cornea.	Assess corneal condition over time and facilitates tracking of changes across the entire cornea Indirect indications of presence of fungal pathogen, precluding species identification Sensitivity: Up to 85% [51] Limited availability

Abbreviations: BA, blood agar; SDA, Sabouraud dextrose agar; CA, chocolate agar; KOH, potassium hydroxide; CFW, Calcofluor white; PCR, polymerase chain reaction; MALDI-ToF MS, matrix-assisted laser desorption/ionization time of flight mass spectrometry; PMF, peptide mass fingerprint.

## Data Availability

Data are contained within the article.
